# Isolation, Characterization of Pyraclostrobin Derived from Soil Actinomycete *Streptomyces* sp. HSN-01 and Its Antimicrobial and Anticancer Activity

**DOI:** 10.3390/antibiotics12071211

**Published:** 2023-07-20

**Authors:** Halaswamy Hire Math, Sreenivasa Nayaka, Muthuraj Rudrappa, Raju Suresh Kumar, Abdulrahman I. Almansour, Karthikeyan Perumal, Girish Babu Kantli

**Affiliations:** 1 Post Graduate Department of Studies in Botany, Karnatak University, Dharwad 580003, Karnataka, India; halaswamy@kud.ac.in (H.H.M.); rmuthuraj@kud.ac.in (M.R.); 2 Department of Chemistry, College of Science, King Saud University, P.O. Box 2455, Riyadh 11451, Saudi Arabia; sraju@ksu.edu.sa (R.S.K.); almansor@ksu.edu.sa (A.I.A.); 3 Department of Chemistry and Biochemistry, The Ohio State University, 151 W. Woodruff Ave, Columbus, OH 43210, USA; perumal.11@osu.edu; 4 Department of Life Sciences, PIAS, Parul University, Vadodara 391760, Gujarat, India; gireesh.k18684@paruluniversity.ac.in

**Keywords:** *Streptomyces* sp., pyraclostrobin, NMR, antimicrobial activity, anticancer activity

## Abstract

The present study demonstrated the isolation, characterization, and antimicrobial and anticancer activity of active metabolite produced from mining-soil-derived actinomycetes. Among the 21 actinomycete isolates, the isolate HSN-01 exhibited significant antimicrobial activity in primary screening and was identified as *Streptomyces* sp. through 16S rRNA gene sequencing. The active metabolite was separated, purified, and confirmed through UV–Vis spectroscopy, FTIR, HR-ESI-MS, and NMR analysis and identified as pyraclostrobin. Further, the active metabolite pyraclostrobin was tested for antimicrobial and anticancer activity against the hepatocellular carcinoma (HepG2) cell line. The metabolite exhibited maximum antimicrobial potential with 17.0, 13.33, 17.66, 15.66, 14.66, and 14.0 mm of inhibition against *B. cereus*, *S. aureus*, *E. coli*, *P. aeruginosa*, *S. flexneri*, and *C. glabrata*. The active metabolite exhibited dose-dependent anticancer potential against the hepatocellular carcinoma (HepG2) cell line with the IC_50_ 56.76 µg/mL. This study suggests that *Streptomyces* sp. HSN-01 is an excellent source of active secondary metabolites with various biological activities.

## 1. Introduction

Emerging diseases and multidrug-resistant bacterial pathogens can be effectively controlled by isolating and identifying beneficial microorganisms that produce bioactive secondary metabolites. The distribution of actinomycetes is diverse and plays a vital role in the degradation of organic matter. Moreover, actinomycetes are considered reservoirs of many secondary metabolites [1]. Secondary metabolites associated with actinomycetes are involved in an organism’s growth and development. It was recorded that about 50% of bioactive metabolites are derived from actinomycetes and remarkably account for antimicrobial, antibiotics, antifungal, and anticancer agents [2]. In line with the novelty of actinomycetes, much attention has been focused on isolating actinomycetes from unexplored regions over the past two decades.

Among the different classes of actinomycetes, the genus *Streptomyces* has been recognized for diverse and novel secondary metabolites. According to a study, around 100,000 antibiotics (70% to 80% of the total antibiotics) were produced by *Streptomyces*, accounting for their promising agrochemical and pharmacological applications (antimicrobial and anticancer). In previous studies, *Streptomyces* species were recorded for their ability to produce antimicrobial compounds belonging to different classes, such as alkaloids, macrolides, peptides, and anticyclones. Some of the *Streptomyces* species, like *Streptomyces nodosus*, *Streptomyces pacificus*, *Streptomyces* RAUACT-1, and *Streptomyces lincolnensis*, are noted for their secretion of antimicrobial compounds such as aureomycin, 10-anthraquinone, lajollamycin, mitomycin, lincomycin, etc. [3]. The different Streptomyces spp. isolated from the soil exhibited synergetic antimicrobial potential against *Staphylococcus aureus*, *Candida albicans*, *Escherichia coli*, and *Bacillus cereus* [4]. In another study, *Streptomyces actinomycinicus* PJ85 showed effective inhibitory action against methicillin-resistant *Staphylococcus aureus* [5]. Earlier studies also reported the significant antimicrobial activity of *Streptomyces filamentosus* strain KS17 and *Streptomyces* sp. KF15 against *Enterococcus faecalis*, *Klebsiella pneumoniae*, *Bacillus cereus*, *Shigella flexneri*, *Escherichia coli*, *Staphylococcus aureus*, *Bacillus subtilis*, *Candida albicans*, and *Candida glabrata* [6,7]. Based on the literature, the *Streptomyces* genus has undoubtedly become an excellent source of new antimicrobial metabolites that can serve as drugs against multidrug-resistant pathogens.

Cancer is the most devastating cause of death in humans, with the highest mortality rate around the world. According to a study, in 2020, nearly 10.0 million deaths and 19.3 million new cases were registered. Among the different cancers, liver cancer is one of the leading cancers that has become a serious health problem affecting humans around the globe. A World Health Organization (WHO) survey showed 780,000 deaths per year and more than 840,000 new cases registered for hepatocellular carcinoma around the globe [8]. Due to its severity, hepatocellular carcinoma (HCC) is the fourth leading cause of death in humans and is predominant in most developing countries [9]. Approximately 700,000 deaths and around 750,000 new cases are reported per year, and over 75% of incidents are reported in Asian countries [10,11]. However, chemical-based treatments for cancer have several side effects and high costs. To overcome the negative effects of chemical treatments, using natural products produced from different actinomycetes can be an excellent alternative that is inexpensive, non-toxic, and has fewer side effects, representing a major strategy that has become of great interest in the research community.

Interestingly, *Streptomyces* species are recognized for their anticancer potential against different cancer cell lines. In a previous report, *Streptomyces* sp. SCSIO 40063 and *Streptomyces tunisiensis* W4MT573222 exhibited anticancer activity against MCF-7, SF-268, PANC-1, and A-549 [12,13]. *Streptomyces atrovirens* (MS5) and *Streptomyces labedae* (MR15) showed cytotoxicity against colon cancer (HCT-116) and hepatocellular carcinoma (HepG2) cell lines. In another study, *Streptomyces* sp. SH8, SH10, and SH13 exhibited anticancer potential against HepG2 and MCF-10A cancer cell lines [14]. Rudrappa et al. [15] reported significant cytotoxicity of *Streptomyces* sp. strain MR28 against melanoma cells.

An active ingredient in a pesticide that belongs to the group of fungicides is pyraclostrobin, which is a pyrazole derivative and inhibits the enzyme activities that are necessary to synthesize fungal cell walls. In addition, pyraclostrobin has synergistic effects with other chemicals and works in a synergistic way with them, including quinones, phenylacetic acids, and sulfur compounds. Also, in vitro tests of pyraclostrobin showed the matrix effect of the drug on solid tumors and the mitochondrial membrane potential as well. Physiological effects, as well as toxicity, can be studied using this product in plant science research [16].

This study aimed at the extraction, isolation, and purification of potent metabolites produced by *Streptomyces* sp. HSN-01, and its biological activities were assessed.

## 2. Results

### 2.1. Isolation and Screening

In this work, 21 actinomycete isolates were isolated from mining soil samples of the Sandur region, Karnataka, India. The actinomycete isolates were then screened for antimicrobial activity against human pathogens, and the HSN-01 was shown to have the most potential among all isolates (Table 1). This indicated the organism’s capacity to produce antimicrobial compounds. Thereby, the isolate HSN-01 was selected for further studies.

### 2.2. Phylogenetic Analysis of Streptomyces *sp.* HSN-01

**Figure 1:**
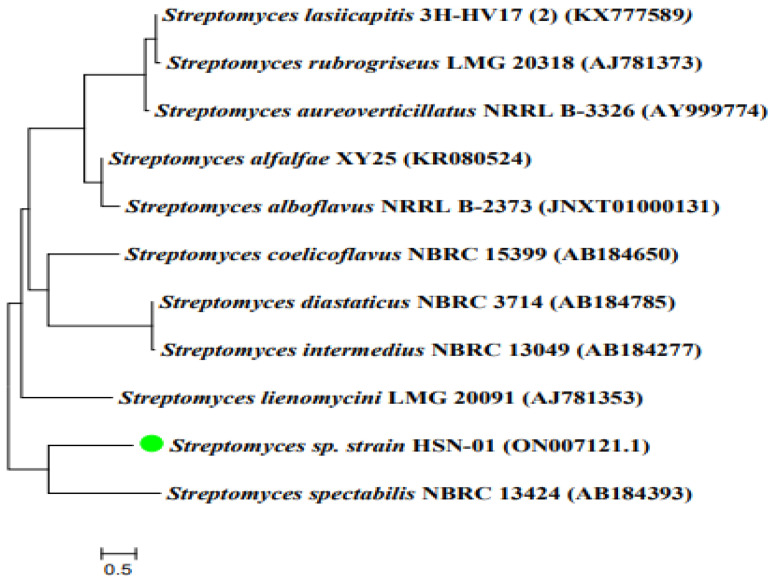
Dendrogram indicating the phylogenetic relation of the isolate HSN-01 with closely related *Streptomyces* species.

The phylogenetic analysis of *Streptomyces* sp. HSN-01 was accomplished by sequencing the 16S rRNA gene. The obtained sequence was 1448 bp in length and deposited in NCBI with the accession number ON007121.1. During the BLAST analysis using EZBioCloud software, the sequence revealed high similarity (98.7%) with the relative sequence of *Streptomyces spectabilis* NBRC13424 (AB184393). Type strain sequences were then retrieved, and a phylogenetic tree was constituted based on the neighbor-joining method (Figure 1). The phylogenetic tree revealed the ancestral relationship of *Streptomyces* sp. HSN-01 with *Streptomyces spectabilis* NBRC13424 (AB184393), forming a single clad.The green color dot indicated in Figure 1 represented isolated organism HSN-01.

### 2.3. Morphological, Physiological, and Biochemical Characterizations of Streptomyces *sp.* HSN-01

**Figure 2:**
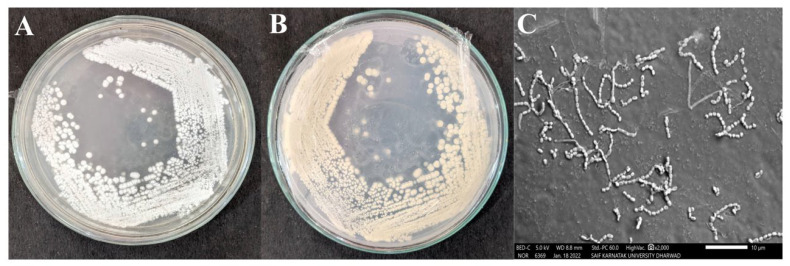
Colony morphology; (**A**) aerial mycelium, (**B**) substrate mycelium, and (**C**) SEM image showing spore chains and spore surface of *Streptomyces* sp. HSN-01.

The *Streptomyces* sp. HSN-01 was sub-cultured on starch casein agar (SCA) medium for morphological analysis. The colonies were flattened, dry, and powdery, having filiform margins. The organism was Gram-positive and showed whitish-colored aerial (Figure 2A) and pale-yellow-colored substrate mycelia (Figure 2B), which often produced yellow-colored soluble pigment (Table 2). SEM analysis of the organism revealed branched fragmenting mycelia containing coliform-shaped spores (Figure 2C).

During physiological characterizations, the *Streptomuyces* sp. HSN-01 exhibited optimum growth at 30 °C and moderate growth at 35 °C. The organism could not grow at 20 °C, and weak growth was reported at 25, 40, and 45 °C. Luxuriant growth was observed at pH 7.0 and weak growth was noticed at pH 8.0; however, no growth was reported at pH 5.0, 6.0, 9.0, or 10.0 (Table 2).

The organism *Streptomyces* sp. HSN-01 revealed diverse biochemical characteristics through VITEK2 analysis, and the results are documented in Table 3. The organism showed 14 positive and 32 negative results out of 46 tests. Enzyme activities like BETA-XYLOSIDASE, L-Lysine-ARYLAMIDASE, Leucine ARYLAMIDASE, etc., were positive for this organism. The organism could utilize D-GLUCOSE as a carbon source and it showed resistance to KANAMYCIN, OLEANDOMYCIN, and POLYMIXIN-B.

### 2.4. Extraction of Secondary Metabolites

*Streptomyces* sp. HSN-01 broth was extracted with an equal volume of ethyl acetate three times. The combined organic phase was then concentrated in vacuo to obtain a brown gum of crude extract (50 mg).

### 2.5. Purification of Ethyl Acetate Extract

The ethyl acetate extract (EtoAc-Ex) was purified by normal-phase column chromatography, and 12 fractions were collected. Of these, three fractions (F5-F7) showed antibacterial activity against *B. cereus* and *E. coli*. The active fractions were then pooled together and further purified through RP-HPLC, and four fractions were obtained. Each fraction was subjected to antibacterial activity, and fraction 2 was active against *B. cereus* and *E. coli*, and the fraction was analyzed further for identification.

### 2.6. Identification and Structure Elucidation

**Figure 3:**
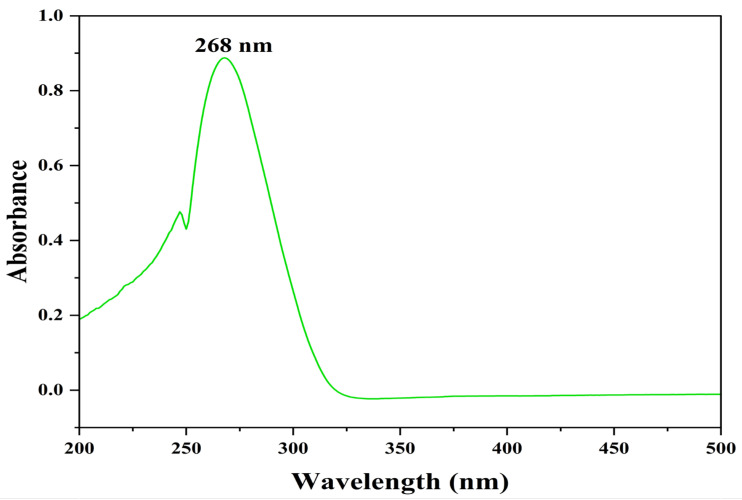
UV–Vis spectrum of pyraclostrobin.

The identification and structure elucidation of the isolated compound were performed by FTIR, HR-ESI-MS, and NMR analysis. The compound was extracted as a pale-yellow crude extract with an unpleasant odor and was found to be completely soluble in DMSO, methanol, and water. The UV absorption spectrum of the compound in MeOH was reported at 268 nm, indicating a polyunsaturated fatty acid group (Figure 3).

**Figure 4:**
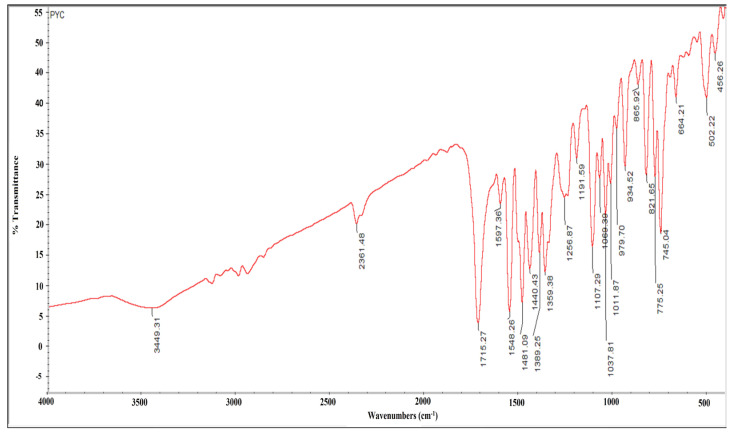
FTIR spectrum of pyraclostrobin isolated from *Streptomyces* sp. HSN-01.

The FTIR spectrum of the compound produced numerous distinct bands at 3449, 2361, 1715, 1597, 1548, 1481, 1440, 1389, 1359, 1256, 1191, 1011, 979, 934, 865, 821, 775, 745, 774, 502, and 456 cm^−1^. The sharp peaks at 3449 and 2361 cm^−1^ indicated O-H stretching alcohol and N-H stretching amine groups. The peak at 1715 cm^−1^ was assigned to the ɑ, ß- unsaturated ester. The 1548 cm^−1^ peak indicated the nitro compound, the 1481 and 1440 cm^−1^ peaks corresponded to alkane and carboxylic acid groups, and the medium-intensity peaks at 1389 and 1359 cm^−1^ correlated to aldehyde and alcohol. The sharp, strong-intensity peaks at 1191, 1107, and 1069 cm^−1^ were associated with primary, secondary, and tertiary alcohol groups. The strong peaks at 1037 and 1011 cm^−1^ belonged to the C-F stretching fluoro compound. The medium-intensity peaks at 979 and 934 cm^−1^ were associated with C=C bending alkene derivatives. However, C-H bending, C-Cl stretching, and C-I stretching halo compounds with 1,4 and 1,2-di-substituted groups were associated with strong-intensity peaks at 865, 821, 775, 745, 664, and 502 cm^−1^, respectively (Figure 4).

**Figure 5:**
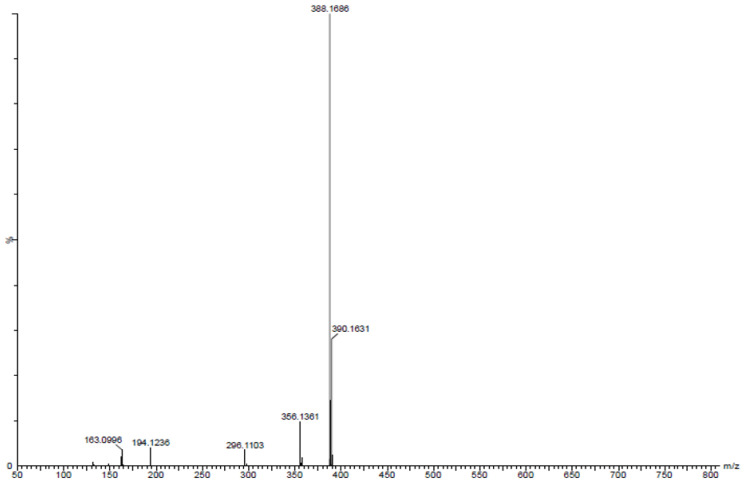
HR-ESI-MS spectrum of pyraclostrobin showing a molecular peak at *m*/*z* 388.1686 [M+H]^+^.

**Figure 6:**
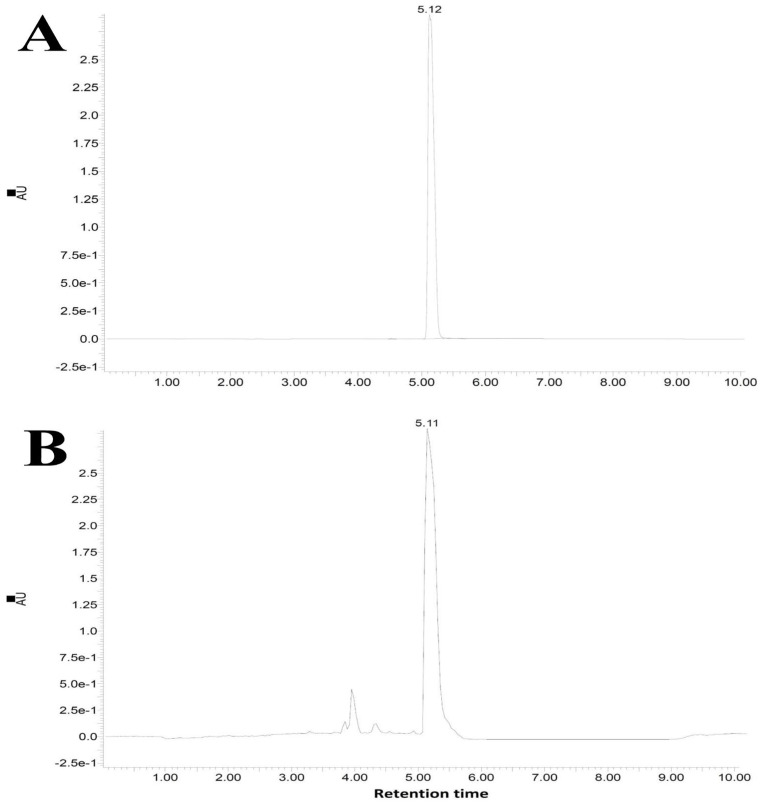
Elusion profiles; (**A**) standard pyraclostrobin (tR = 5.12 min) and (**B**) pyraclostrobin from *Streptomyces* sp. HSN-01 (tR = 5.11 min).

The mass analysis of the compound showed the prominent molecular ion [M+H]^+^ peak at *m*/*z* 388.1686, yielding the accurate mass of 387.0985 (Figure 5). The comparison of the retention time of the standard and compound revealed similar retention times of 5.12 and 5.11 min, respectively (Figure 6A,B).

**Figure 7:**
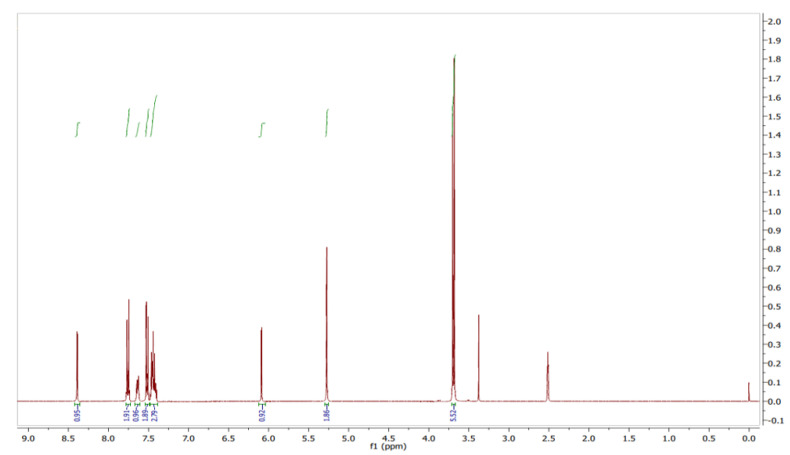
^1^H NMR spectrum of pyraclostrobin.

**Figure 8:**
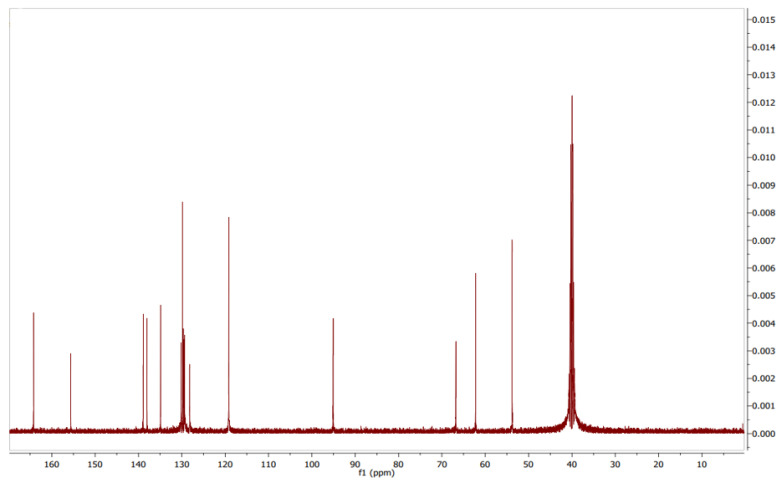
^13^C NMR spectrum of pyraclostrobin.

The ^1^H NMR spectra of the compound were recorded in DMSO-*d_6_* solution using TMS as an internal standard. The distinct proton signals that occurred in the spectrum pointed to a distinct chemical environment. ^1^H-NMR data of the compound showed the following resonance: *δ*, ppm 3.69 (6H, d), 5.27 (2H, s), 6.09 (1H, d), 7.44 (3H, m), 7.52 (2H, m), 7.64 (1H, m), 7.76 (2H, m), and 8.31 (1H, d) (Figure 7). These results suggested the presence of pyrazole moiety. The red color (Figure 7 and Figure 8) indicates the NMR signals and green color (Figure 7) represents integration value of protons.

**Figure 9:**
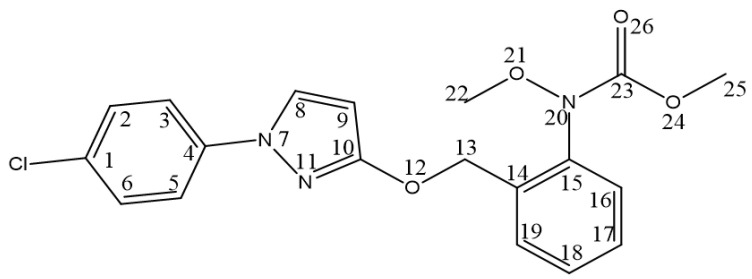
Chemical structure of the compound pyraclostrobin.

The assignment of carbons in the molecular structure was confirmed using ^13^C NMR, which confirmed the presence of 19 carbon signals (Figure 8). The chemical shifts of carbons at *δ_C_* 129.60 (C-8), 95.02 (C-9), and 164.28 (C-10) suggested pyrazole moiety. Additionally, *δ_C_* from 154 to 119 (C-1 to C-6 and C-14 to C-19 and C-23) defined the benzene group in the structure with the amide group, whereas the other aliphatic group was correlated with 6.93 (C-6), 62.23 (C-22), and 53.27 (C-25). Based on these findings, the structure (Figure 9) was determined to be pyraclostrobin.

Therefore, based on the obtained data from UV, FTIR, HR-ESI-MS, and NMR analyses, the structure of the compound was determined to be pyraclostrobin with the chemical formula C_19_H_18_ClN_3_O_4_ (Figure 9).

Pyraclostrobin: pale-yellow-colored amorphous powder; tR = 5.11 min; UV (MeOH) λ_max_ 268 nm; FTIR (KBr)ν_max_ 3449, 2361, 1715, 1597, 1548, 1481, 1440, 1389, 1359, 1256, 1191, 1011, 979, 934, 865, 821, 775, 745, 774, 502 and 456 cm^−1^, ^1^H and ^13^C (400 MHz) NMR data (see Figure 9); HR-ESI-MS *m*/*z* 388.1686 [M+H]^+^, (calcd. for C_19_H_18_ClN_3_O_4_ 387.0985).

### 2.7. Antimicrobial Activity and MIC Assay

**Figure 10:**
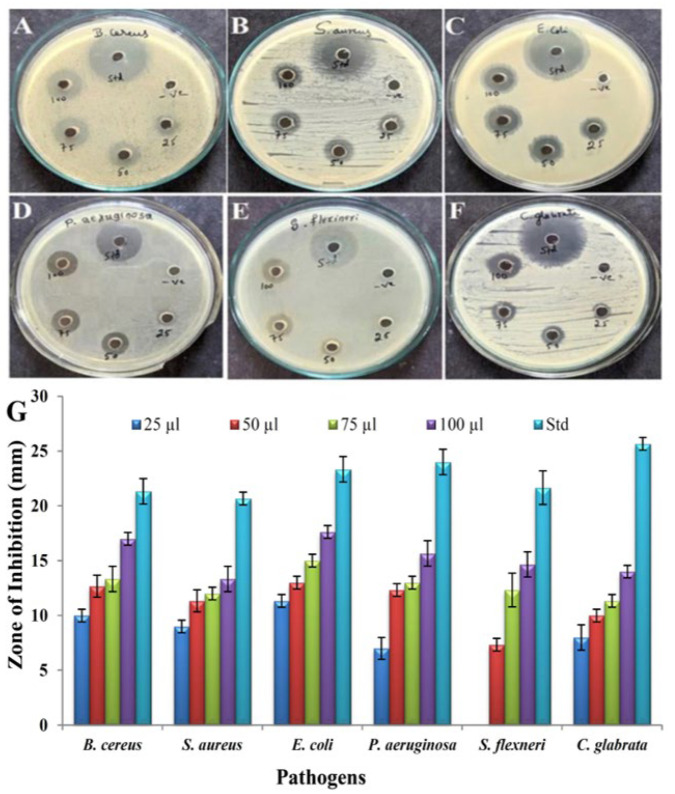
Antimicrobial activity of pyraclostrobin by agar well diffusion method; (**A**) *B. cereus*, (**B**) *S. aureus*, (**C**) *E. coli*, (**D**) *P. aeruginosa*, (**E**) *S. flexneri*, (**F**) *C. glabrata.* (**G**) Bar graph showing zones of inhibition of pathogens at different concentrations of pyraclostrobin.

The antimicrobial activity of pyraclostrobin was tested against two Gram-positive and three Gram-negative pathogens and one yeast strain. The antimicrobial activity was assayed with 25–100 µL of purified compound through the agar well diffusion method (Figure 10A–F). The maximum inhibition zone was recorded against *E. coli* (11 to 17.66 mm) and the minimum activity against *S. flexneri* (7.33 to 14.66 mm), shown in the bar diagram (Figure 10G). However, the compound could inhibit the growth of all the pathogens. The MIC value of the tested pathogens is shown in Table 4. The pathogen *S. aureus* showed the lowest MIC value of 1.6 µg/mL, and the highest MIC value of 25 µg/mL was recorded against the pathogen *P. aeruginosa*. DMSO was used as a control and showed no activity.

### 2.8. Evaluation of Anticancer Activity

**Figure 11:**
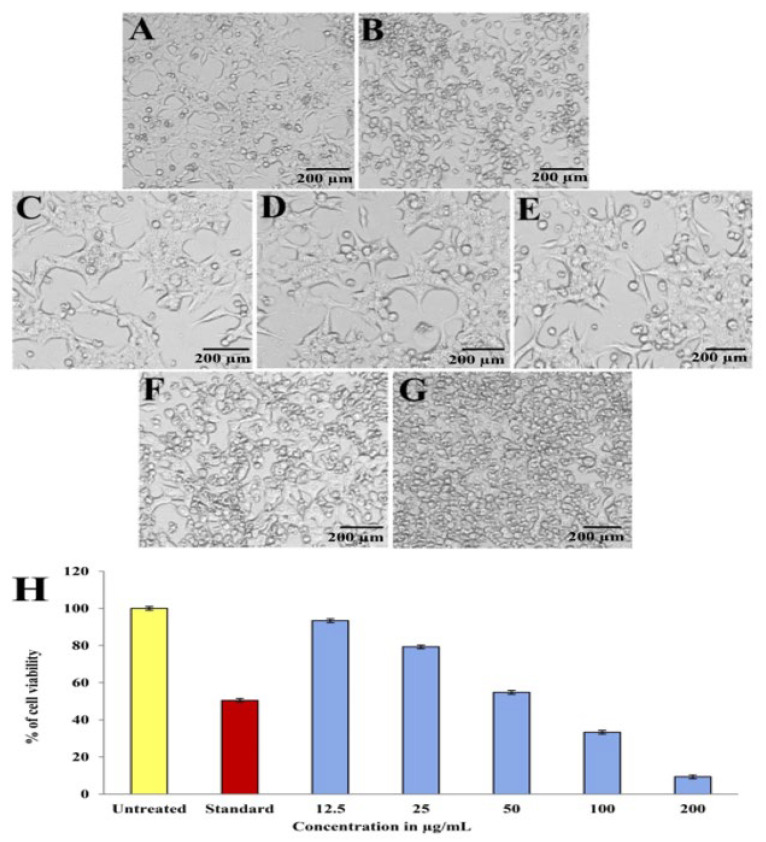
In vitro anticancer activity of purified compound at different concentrations against HepG2 cell line; (**A**) untreated, (**B**) standard control (camptothecin), (**C**) 12.5 µg/mL, (**D**) 25 µg/mL, (**E**) 50 µg/mL, (**F**) 100 µg/mL, (**G**) 200 µg/mL. (**H**) Comparative % cell viability of HepG2 cells treated with different concentrations of purified compound (scale bar: 200 µm).

As the concentration of purified compound increased, the anticancer activity of the compound, which was evidenced by the distinct changes in shape, size, and other morphological details of the tumor cells, demonstrated a progressive decline in growth and inhibited the growth of human hepatoblastoma (HepG2) cells (Figure 11A–G). In vitro anticancer activity of the purified compound was tested against the HepG2 cell line and was evaluated and compared with no treatment and treatment with the standard drug camptothecin (10 µM/mL). The critical result obtained by MTT assay in the HepG2 cell line exhibited a concentration-dependent decrease in viability. The cell viability was recorded as 93.39%, 79.30%, 54.77%, 33.31%, and 9.31% at 12.5, 25, 50, 100, and 200 µg/mL concentrations of pyraclostrobin (Figure 11H). It was determined that pyraclostrobin has an IC_50_ value of 56.76 µg/mL against HepG2 cells, which suggests that it can inhibit the proliferation of cancer cells in vitro. Comparing the treated HepG2 cells with the healthy ones, we noticed deformed cells, blebbing of the membrane, and the release of a matrix.

## 3. Discussion

Actinomycetes from understudied habitats, particularly those in mining sites, constitute a potentially valuable source of bioactive compounds. The exploration of harsh environments for isolation results in finding novel metabolite-producing microbes. The current study aimed to explore mining soils to isolate actinomycetes. Actinomycetes isolated from such ecosystems found at mining sites can be used in biological applications and constitute a potentially important source of bioactive compounds [17]. Sarika et al. [18] collected 28 soil samples from coal mining sites to isolate antimicrobial-producing actinomycetes. Sapkota et al. [19] isolated 41 actinomycetes from soil samples at altitudes of 1500 to 4380 m from Nepal. Various types of actinomycete isolation media were used for the isolation; among them, SA, SCA, and ISP [20,21,22] showed good media conditions for the growth of the isolates. Bhat et al. [7] isolated 31 actinomycetes from cave soil using SCA medium, Rudrappa et al. [23] isolated 40 actinomycete isolates from soil samples using SCA medium, and Chakraborty et al. [24] isolated 70 actinomycetes strains from marine sediment soil samples using SA, ISP, and AIA medium; among them, SA exhibited the highest number of colony-forming units. In the present study, 21 isolates were obtained from mining soils. The primary screening of the isolates disclosed that the isolate HSN-01 had the most potential to inhibit the growth of pathogens. The microbial strain of interest was seeded by a single streak in the center of the agar plate. After an incubation period specific to the microbial strain, the plate was seeded with the microorganisms tested by a single streak perpendicular to the central streak. After further incubation, the antimicrobial interactions were analyzed by observing the inhibition zone size. Since the area of each test streak was fixed at 150 mm^2^, it was possible to quantify regions of growth and measure the clear/inhibited areas with a graph paper template. The clear areas for different test species are indicators of antibiotic activity and are proportional to antibiotic concentration and diffusion. To express these results quantitatively and to prepare quantitative antibiotic activity score matrices, the following equation was used:

Percentage area specific differential antibiotic activity score (PASDAAS) = (AWG/TSA) × 100, where AWG is the area on the plate without growth for each test panel streak, and TSA is the total streak area (150 mm^2^) scored for each test panel streak [25].

The cross-streak method was modified as the streak non-uniformity caused a problem with respect to quantifying the results. Moreover, mutual antibiosis due to the use of multiple cultures in the test panel could not be ruled out. The distance between the test streak and the actinomycetal strain was kept constant to reduce the effect of probable mutual antibiosis, as multiple cultures were used in the test panel. Since the test pathogen population was delimited within a defined streaking zone, antibiosis would indicate its impact with respect to that zone and thus, the antibiosis reaction could be easily quantified [26]. At the time the results were observed, the hydrogen ion concentration of the agar adjacent to the streak was determined in order to eliminate inhibition due to acidity alone. On the basis of the results obtained, we can differentiate which organism showed good or moderate activity.

Different media were used to isolate actinomycetes; SCA was proved effective for isolation [27]. Considering data from previous reports on isolate growth and colony-forming capacity, SCA medium was further used in this work. Using SCA medium, we investigated the morphological and physiological characterization of the actinomycete isolates. The isolates’ physical and cultural traits displayed a variety of hues. Actinomycetes were found in various colors across the analyzed soil samples. These colors included chalky white, yellow, orange, violet, green, and gray. The colors of the aerial and substrate mycelium are considered to be instantly recognizable characteristics of actinomycete isolates [28].

The molecular characterization of the potential isolate HSN-01 was performed by 16S rRNA gene sequencing. The analysis showed that the isolate HSN-01 revealed maximum sequence similarity (98.7%) with *Streptomyces spectabilis*. The actinomycete *Streptomyces spectabilis* produces various antibiotics like gentamicin, kanamycin, dihydrospectinomycin, etc. It also exhibits good antimicrobial and anticancer activity [29,30]. In the same way, our isolated organism HSN-01 shares phylogeny with *Streptomyces spectabilis*, exhibits various activities, and produces secondary metabolites. The 16S rRNA gene sequencing is an important criterion for identifying actinomycetes at their species level. Similar results were reported when 16S rRNA gene sequencing and phylogenetic tree analysis were performed on *Streptomyces paradoxus* strain KUASN-7 [31] because of the hyper-variable and conserved regions of the 16S rRNA gene. There are hyper-variable regions that provide species-specific signature sequences that help distinguish bacterial, archaeal, and microbial eukaryote strains, whereas conserved regions provide universal primer binding sites [32]. In the current study, the isolate HSN-01 exhibited prominent characteristics (morphological and molecular) of *Streptomyces* and was considered as *Streptomyces* sp. HSN-01.

The morphological and physiological characterization of HSN-01 was conducted on SCA medium. HSN-01 exhibited sporulation and chalky white, dry, powdery colonies with a crowded filamentous appearance with aerial mycelium. All the isolates exhibited sufficient growth on SCA medium, and each isolate was determined as a Gram-positive organism through branched morphology. A similar study was conducted on the characterization of actinomycetes and found that 25 isolates showed the potential to hydrolyze starch and casein [33]. In another study, Almaki [34] reported that among eight isolates, the *Streptomyces* SCA-7 exhibited white aerial mycelium and showed the production of diffusible pigment on SCA medium. Different media like SA, SCA, AIA, and ISP were used to isolate actinomycetes; SCA was proved effective for isolation [27]. Considering previous findings on isolate growth and colony-forming capacity, SCA medium was further used in this work. Siddarth et al. [35] reported that four strains did not produce any diffusible pigment among five isolated *Streptomyces*, and that *Streptomyces* sp. S1A and *Streptomyces* sp. SCA35 exhibited a powdery colony and white aerial mycelium. Our study’s results indicated optimum growth at a pH of 7.0. Actinomycetes can withstand a wide range of temperatures. In our study, the optimal temperature for the growth and production of antimicrobial metabolites was determined to be 30 °C, confirming that the organism was a strict mesophile. Similar studies were carried out by other researchers, who reported that optimal pH and temperature are essential for the growth and metabolism of actinomycetes [36,37]. The extraction of metabolites from the culture broth of HSN-01 was carried out using ethyl acetate and exhibited varied results against the tested pathogenic organisms. Numerous studies have suggested that ethyl acetate is the best solvent for extracting bioactive compounds (antimicrobial and anticancer), particularly from the genus *Streptomyces* [38].

In the current study, the identification and structure elucidation of the isolated compound were realized by UV, FTIR, HR-ESI-MS, and NMR analysis. The compound was extracted as a pale-yellow sticky residue with an unpleasant odor and was found to be completely soluble in DMSO, methanol, and water. UV–Vis spectroscopy was used for quantitative analysis. Maximum absorbance peaks ranged between 215 and 270 nm, and the characteristics of absorption peaks indicated a highly polyene nature. In general, strobilurin derivatives are strong chromophores in the UV range. Natural substances can also be determined using UV–Vis spectroscopy. The FTIR spectra of the active actinomycete strain HSN-01 revealed numerous peaks. The peaks were used to define related groups. The peaks represent functional groups like alkyl halides, aromatic, alkenes, carboxylic acid, different alcoholic groups, di-substituted compounds, esters, and different halo compounds. The Gram-positive *Streptomyces* are the most studied and well-known group of bacteria that can produce the most important secondary metabolites—similar reports about the FTIR analysis of ethyl acetate extracts from *Streptomyces* sp. CRB46 [39] and *Actinomycetes* sp. [40] suggested the presence of various functional groups such as alkanes, amines, phenols, carboxylic acids, and aromatic compounds. Through FTIR spectroscopy, this study illustrates the potential of *Streptomyces* species in synthesizing antibiotics and further examines the antibacterial activity of diverse isolates. In the future, this study could be helpful in the fight against drug-resistant illnesses [41].

In order to bring up economically valuable compounds for natural product discovery, it is imperative to study the crude extract from *Streptomyces* sp. HSN-01, in detail, in order to demonstrate its purification and characterization, identify its constituent active secondary metabolites, and confirm their identity. An SCA medium was fermented using the strain HSN-01, resulting in the extraction and concentration of the cell-free culture filtrate and the purification and characterization of crude compound. The commonly used extraction solvents include acetonitrile, dichloromethane, methanol, chloroform, etc., and the purifiers include PSA and C-18 [42]. A rapid method for the analysis of compounds in organisms was established by changing extraction solvents and purifiers and comparing the extraction rates, based on the classical QuEChERS method.

For soil microorganisms, acetonitrile solution was used for extraction, and C-18 had the best purification efficiency. The recovery reached nearly ≤90%, which was much higher than other extraction solvents and purifiers. Thus, pure acetonitrile with 0.1% formic acid was deemed the best extraction solvent, and C-18 was deemed the best purifier for the isolation of compounds in soil microorganisms [43]. The main active compound was identified as pyraclostrobin, which has a molecular formula C_19_H_18_ClN_3_O_4_ + H^+^ and bears resemblance to active compounds reported in databases or the literature (e.g., http://chembiofinder.cambridgesoft.com and www.chemspider.com (accessed on 5 May 2023). Thus, pyraclostrobin is apparently an alkaloid unreported in plants, algae, or actinobacteria.

One of the largest sources of secondary metabolites is *Streptomyces*, producing antibiotics, herbicides, parasiticides, immunosuppressive agents, antitumor compounds, and other compounds of great pharmaceutical and industrial use [44]. As a model prokaryotic system, *Streptomyces* has a complex and multicellular lifecycle. *Streptomyces* has the capability to produce a wide range of secondary metabolites, making it a multicellular system. Although potential strains may produce secondary metabolites at a low level, process optimizations as well as the genetic engineering of biosynthetic pathways are often required to enhance secondary metabolite production [45]. As a result of recent developments in genetic engineering, synthetic biology, mass spectrometry, and cheminformatics, various microorganisms, including *Streptomyces*, have been refactored and screened with precision. As a result of the coupling of advances in genome mining, bioinformatics, and genetic engineering tools, rational metabolic engineering strategies have been developed for *Streptomyces* and natural products have been discovered faster [46]. In this study, a validated QuEChERS and HPLC–MS/MS analytical method of pyraclostrobin was developed. This method had satisfactory parameters of higher purity, accuracy, and precision. The results show that pyraclostrobin from *Streptomyces* was extracted with 0.1% formic acid and acetonitrile and purified with C-18 column. The present study reports that the mining soil actinomycete *Streptomyces* sp. HSN-01 produces a strobilurin compound (pyraclostrobin).

The compound was tested for its antimicrobial activities towards *B. cereus*, *S. aureus*, *E. coli*, *P. aeruginosa*, *S. flexneri*, and *C. glabrata* using the well diffusion method. The results show that pyraclostrobin exhibited a maximum zone of inhibition against *E. coli*, and the smallest zone of inhibition was against *S. flexneri*. In comparison to Gram-positive pathogens, Gram-negative pathogens had better resistance. Gram-positive pathogens showed higher inhibition zones. This could be attributed to the presence of lipopolysaccharides as significant structural units in the outer membrane of Gram-negative bacteria. In contrast, Gram-positive bacteria lack this protective barrier and are susceptible to metabolites [47]. Pyraclostrobin inhibits bacterial growth in several ways, including inhibiting the synthesis of nucleic acids and proteins, altering the permeability of the bacterial cell membrane, causing damage to the membrane and cell wall, inhibiting bacterial metabolism, and inhibiting efflux pumps. The bioactive ansamycins produced by *Streptomyces* sp. from a hyper-arid desert exhibit good antibacterial activity against *S. aureus* [48]. The MICs of pyraclostrobin against pathogenic bacteria were lower against *S. aureus* when compared to standard antibiotic streptomycin. It was evident from the MIC results that the Gram-positive pathogens were susceptible to lower concentrations of the compound, and the Gram-negative pathogens were susceptible to higher concentrations.

The compound showed a broad spectrum of anticancer potential against the human liver cancer (HepG2) cell line. A dose-dependent action against the HepG2 cell line showed varying inhibition percentages according to the increase in the concentration (IC_50_ 56.76 µg/mL) of the extract. The obtained result agrees with another study where *Streptomyces* sp. RMS518F exhibited anticancer potential against the human colon carcinoma (HCT-116) and the hepatocellular carcinoma cell line (HepG2) with IC_50_ values of 73.4 and 62.8 µg/mL, respectively [49]. Many reports suggested that the cytotoxicity is due to the bioactive metabolites in the extract that facilitate the interaction with DNA regions with high G-C content, inhibition of the activity of topoisomerase-II, and breaking of single-stranded DNA [50]. In the present study, the different metabolites identified in the HSN-01 extract might be involved in suppressing the HepG2 cell line. The results were strongly supported by the study by Bhat et al. [7], where different metabolites were identified in the ethyl acetate extract of *Streptomyces* sp. KF-15 exhibited potential anticancer action against the HeLa cell line.

## 4. Materials and Methods

### 4.1. Pathogens Used in the Study

The pathogenic microbial strains, namely, *Bacillus cereus* (*B. cereus*) (MTCC 11778), *Staphylococcus aureus* (*S. aureus*) (MTCC 6908), *Escherichia coli* (*E. coli*) (MTCC 40), *Pseudomonas aeruginosa* (*P. aeruginosa*) (MTCC 9027), *Shigella flexneri* (*S. flexneri*) (MTCC 1457), and *Candida glabrata* (*C. glabrata*) (MTCC 3019), were procured from the Institute of Microbial Technology (IMTECH), Chandigarh, India. The chemicals and media used in the study were purchased from Hi-Media Laboratories Pvt Ltd. Thane, (West) 400604, Maharashtra, India, and Sigma-Aldrich Chemicals Private Limited, Bangalore, India.

### 4.2. Isolation and Screening

The soil samples were collected from the Sandur mining region (15°01′49.8″ N, 76°55′17.7″ E) at a depth of around ~15 cm using a sterile spatula and preserved at 4 °C in sterile polythene bags. The actinomycetes were obtained using the conventional serial dilution procedure on starch casein agar (SCA) (#M801, Hi-media, Hi-Media Laboratories Pvt Ltd. Thane, (West) 400604, Maharashtra, India) and incubated for 12 to 14 days at 30 ± 2 °C. The isolates were later purified and preserved on starch casein agar (SCA) medium at 4 °C [51]. Primary screening of the actinomycete isolates was performed by the cross-streak method against the pathogens *B. cereus*, *S. aureus*, *E. coli*, *P. aeruginosa*, *S. flexneri*, and *C. glabrata*. The actinomycete isolates were grown as straight lines in the middle of separate Petri plates on ISP2 medium (#M424, Hi-Media) at 30 °C for 7 days. After incubation, the pathogens were streaked at a 90° angle to the actinomycete streaks and incubated for 24 h at 37 °C. Further analysis was carried out with the organism that showed the highest inhibition against pathogens.

### 4.3. Molecular Characterization

The molecular level identification of isolate HSN-01 was determined by sequencing the 16S rRNA gene. The genomic DNA was extracted using the HipurA *Streptomyces* DNA purification kit (#MB527, Hi-media, Hi-Media Laboratories Pvt Ltd. Thane, (West) 400604, Maharashtra, India) in accordance with the manufacturer’s guidelines. The universal primers 27F and 1492R were utilized to perform PCR, which was programmed as follows: amplification of 35 cycles at 94 °C for 45 s, annealing at 55 °C for 60 s, and extension at 72 °C for 60 s. The cycle was repeated 30 times, and the final extension was carried out at 72 °C for 10 min. Later, PCR amplicons were validated by electrophoresis with 1 kb of the reference DNA ladder. The DNA analyzer was used to sequence the desired PCR product, and the sequence was submitted to the gene bank through the National Centre for Biotechnology Information website. BLAST analysis was performed to ascertain the phylogenetic neighbors of the isolate using the Ez-Bio-cloud platform (https://www.ezbiocloud.net (accessed on 1 June 2023). Finally, an evolutionary tree was constructed with similar strains using MEGA7.0 software [52].

### 4.4. Morphological, Physiological, and Biochemical Characterizations of Streptomyces *sp.* HSN-01

Morphological, physiological, and biochemical characterizations of *Streptomyces* sp. HSN-01 were determined according to the method of Chakraborty et al. [6]. Pigmentation was recorded for morphological characterizations, colony characteristics, and the color of aerial and substrate mycelia. To analyze the spore chain and spore surface morphology, the *Streptomyces* sp. HSN-01 was studied with a scanning electron microscope (SEM) (JSM-IT500, JEOL, Musashino, Tokyo, Japan). Physiological characterization was performed by growing the isolate from different media like starch casein agar (SCA), starch agar (SA), actinomycetes isolation agar (AIA), and international streptomyces agar (ISP) at various temperatures ranging from 20 to 40 °C and pH ranging from 5.0 to 10.0. Biochemical properties were studied using the Vitek-2 BCL card test kit from BioMerieux (BioMerieux SA, 85 Voie Romaine, 69290, Craponne, France).

### 4.5. Fermentation and Extraction

In the first stage, *Streptomyces* sp. HSN-01 was inoculated as seed culture in 50 mL of starch casein broth and grown for 7 days at 30 °C. In the second stage, 3 L of starch casein broth in an Erlenmeyer flask was inoculated with 30 mL of seed culture and incubated for 20 days at the same temperature on a shaking incubator (150 rpm). After the complete growth of the organism, the biomass was separated by centrifugation at 5000 rpm for 15 min. To extract secondary metabolites, ethyl acetate solvent in a 1:1 (*v*/*v*) ratio was mixed with the supernatant, shaken intermittently, and left for 24 h for proper extraction of metabolites. The organic layer was separated and concentrated at 40 °C under low pressure using an IKA RV-8 rotary evaporator (IKA, Staufen, Germany).

### 4.6. Purification and Characterization

The concentrated ethyl acetate extract (EtoAc-Ex) was then subjected to column chromatography (35 × 1.0 cm) packed with silica gel (60–120 mesh size, #GRM7477, Hi-media). The fractions were eluted with an increasing polarity gradient solvent system of dichloromethane (DCM) and methanol (MeOH) (100:0, 80:20, 60:40, 40:60, 20:80, 0:100, *v*/*v*) to afford 12 fractions. Each fraction was examined for antibacterial activity against *B. cereus* and *E. coli*. The active fraction was pooled and purified again using reverse phase-high performance liquid chromatography (RP-HPLC) and some modified methods [42]. Elution was carried out with a solvent system of 70% isocratic methanol and water at a flow rate of 1 mL/min and at a wavelength range of 200 to 400 nm to yield a compound. The purified fraction was concentrated and checked for antibacterial activity against *B. cereus* and *E. coli.*

### 4.7. Identification of the Compound

The purified bioactive compound was analyzed by UV to inspect the wavelength absorbed by the compound using a Jasco V-670 UV-Vis Spectrophotometer (JASCO corporation, 2967-5 Ishikawa-machi, Tokyo, Japan). To carry out the FTIR analysis, thin discs were made with KBr, and the infrared spectrum of the compound was recorded using NICOLET 6700, Thermo Fisher Scientific, Waltham, MA, USA. High-resolution electrospray ionization mass spectrometry (HR-ESI-MS) was performed using the Xevo G2-XS QTOF mass spectrometer (Waters Corporation, Milford, MA, USA). The instrument was equipped with an Accucore C-18 column (2.6 µm, 50 × 4.6 mm) and the temperature of the column was maintained at 40 °C. The mobile phase consisted of 0.1% formic acid and acetonitrile and was used to run the standard (#H4530, Sigma-Aldrich Chemical Private Limited, Bangalore, India) and the purified compound from *Streptomyces* sp. HSN-01 to compare their retention times. The chromatographic is in the range of 50 to 400 *m*/*z*. Nuclear magnetic resonance (NMR) spectroscopy was performed using an FT-NMR spectrometer (400 MHz, JNM-ECZ 400S, JEOL, 11 Dearborn Road, Peabody, MA, USA). ^1^H NMR and ^13^C NMR spectra were recorded. Trimethylsaline was used as an internal standard, and the chemical shifts of the compound were recorded in Deuterated DMSO (DMSO-*d6*). Spin multiplets were reported as s (singlet), d (doublet), and m (multiplet).

### 4.8. Antimicrobial Activity and MIC Assay

Antimicrobial assay of the purified compound from *Streptomyces* sp. HSN-01 was performed against six pathogenic microorganisms using the agar well diffusion method. The working solution of the compound was prepared in DMSO (1 mg/mL), and the antimicrobials like streptomycin and amphotericin-B (1 mg/mL each) were used as a positive control. The pathogens were freshly cultured, and 0.5 McFarland concentrations (1.5 × 10^8^ UFC/mL) were used for the antimicrobial assay. Mueller–Hinton (MH) agar (pH 7.4) plates were prepared and inoculated separately with 100 µL of test pathogens. Then, 6 mm wells were made, and the respective wells were filled with final concentrations (25–100 µg/µL) of 250, 500, 750, and 1000 µg of the compound dissolved in DMSO. Dimethylsulfoxide (DMSO) was employed as a negative control. The plates were incubated for 24 h at 37 °C. The antimicrobial assay was repeated thrice, and each pathogen’s mean zone of inhibition was calculated [6,7].

The minimum inhibitory concentration (MIC) of the pathogens was carried out according to the method of the Clinical and Laboratory Standards Institute (CLSI). The compound in a concentration of 100 µg/mL was prepared in DMSO (99.8%). Streptomycin and amphotericin-B (1 mg/mL each) were used as standards and DMSO as a control for this study. The microbial suspension was prepared (0.5 McFarland Standards), and 50 µL of each pathogen was dispensed in wells containing 200 µL of MH broth in 96-well plates. Then, 100 µL of the purified compound and positive controls was pipetted out for pathogens and serially diluted twofold up to column 11. Column 12 alone was designated as a sterility control, and the plates were incubated at 37 °C for 24 h. After the incubation, 30 µL of resazurin (0.015%) was added to each well and incubated for 2 to 4 h for the observation of color change. A change in color from purple to pink indicated a positive response, and the lowest concentration at which a color change was noted was the MIC value.

### 4.9. Evaluation of Anticancer Activity by MTT Assay

The principle involved in the MTT assay is the reduction of tetrazolium salt into blue-colored formazan by the enzyme succinate dehydrogenase. The hepatocellular carcinoma (HepG2) cell line was collected from NCCS, Pune. The HepG2 cells were sub-cultured with 10% FBS and maintained at 37 °C for 24 h in a 5% CO_2_ atmosphere. Assays were conducted with medium without cells, control (DMSO) with cells but test compound, and standard with cells and 10 µM camptothecin. At a cell density of 20,000 cells/well, HepG2 cells were poured into 96-well flat-bottom plates for 24 h. The cells were treated with 12.5–200 µg/mL concentrations of a purified compound for 24 h, followed by 0.5 mg/mL of reagent. After incubating, a gyratory shaker was used to gently shake, stir, and dissolve the MTT product in DMSO. The plates were encapsulated in aluminum foil and incubated for 3 h. Absorbance was recorded on an ELISA reader at 570 nm as a standard reference [53]. The percentage of inhibition was calculated, and the concentration of extract needed to inhibit cell growth by 50% (IC_50_) was determined by the dose–response curves for each cell line using the following formula:

% of cell viability = [Absorbance of a sample at 570 nm/Absorbance of control at 570 nm] × 100

### 4.10. Statistical Analysis

The results of at least three independent experiments are shown as means and standard deviations (SDs).

## 5. Conclusions

The *Streptomyces* sp. HSN-01 was isolated from the unexplored Sandur mining region in Ballari, Karnataka, India, and showed significant antimicrobial and antitumor activities. Hence, *Streptomyces* sp. HSN-01 was selected and characterized using optimized media and growth conditions such as pH, temperature, nitrogen, and carbon sources. The natural environment holds significant potential for rare and novel actinobacterial communities, and extensive investigations are required for isolations and creative and efficient taxonomic procedures that may result in the identification of novel genera and novel species of actinomycetes. It is now necessary to look into previously undiscovered environments, particularly those near deep mines, to find new bioactive substances that might benefit humanity. In addition, the compounds analyzed by spectroscopic and chromatographic analysis showed antibacterial and anticancer properties. Other bioassays of isolated compounds are needed in the future. Nevertheless, this study supports the notion that unexplored regions of mining areas hold promising *Streptomyces* isolates with secondary metabolites that could be resourceful in developing safer drugs for several medical and pharmaceutical applications.

## Figures and Tables

**Table 1:** Antimicrobial activity of actinomycete isolates against different test pathogens.

Actinomycete Isolates	*B. cereus*	*S. aureus*	*E. coli*	*P. aeruginosa*	*S. flexneri*	*C. glabrata*
HM-01	+++	+++	−	+	+	+
HM-02	−	++	−	+++	−	++
HM-03	−	+	++	+	+++	−
HM-04	−	+++	−	−	++	−
HM-05	++	−	+	++	−	+
HM-06	−	−	+++	−	+	++
HM-07	++	+++	−	−	−	++
HSN-01	*+++*	*++*	*+++*	*+++*	*++*	*+++*
HSN-02	++	+	−	++	+++	++
HSN-03	−	++	−	+++	−	−
HSN-04	++	−	++	+	+	−
HSN-05	+++	+	++	−	+	++
HSN-06	+	++	−	+	+++	−
HS-01	+++	−	−	++	+	−
HS-02	−	+	−	++	−	++
HS-03	++	−	+	−	+	−
HS-04	+	+++	−	−	++	+++
HS-05	+++	−	+	++	−	+
HS-06	−	−	++	−	+++	−
HS-08	+	−	++	+	+++	+
HS-09	++	−	−	++	−	−

Note: +++ = good activity, ++ = moderate activity, + = weak activity and − = no activity.

**Table 2:** Morphological and physiological characterizations of *Streptomyces* sp. HSN-01.

Morphological Characterizations			
Colony characteristics	Aerial mycelia		Substrate mycelia
Color	Whitish		Pale yellow
Gram staining	Gram-positive		
Margin	Filiform		
Elevation	Elevated		
Texture	Powdery and dry		
Pigmentation	Yellow colored pigment		
**Physiological Characterizations**			
Growth at different temperature		Growth in different pH	
20 °C	−	pH 5.0	−
25 °C	w	pH 6.0	−
30 °C	+++	pH 7.0	+++
35 °C	++	pH 8.0	w
40 °C	w	pH 9.0	−
45 °C	w	pH 10.0	−

Key: +++ = optimum growth, ++ = normal growth, w = weak growth, − = no growth.

**Table 3:** Biochemical characterization of *Streptomyces* sp. HSN-01.

Tests	Results	Tests	Results
BETA-XYLOSIDASE	+	D-MANNITOL	−
L-Lysine-ARYLAMIDASE	+	D-MANNOSE	−
L-Aspartate ARYLAMIDASE	+	D-MELEZITOSE	−
Leucine ARYLAMIDASE	+	N-ACETYL-D-GLUCOSAMINE	−
Phenylalanine ARYLAMIDASE	+	PALATINOSE	−
L-Proline ARYLAMIDASE	−	L-RHAMNOSE	−
BETA-GALACTOSIDASE	−	BETA-GLUCOSIDASE	+
L-Pyrrolidonyl-ARYLAMIDASE	+	BETA-MANNOSIDASE	−
ALPHA-GALACTOSIDASE	−	PHOSPHORYL CHOLINE	−
Alanine ARYLAMIDASE	+	PYRUVATE	+
Tyrosine ARYLAMIDASE	+	ALPHA-GLUCOSIDASE	−
BETA-N-ACETYL-GLUCOSAMINIDASE	+	D-TAGATOSE	−
Ala-Phe-Pro ARYLAMIDASE	+	D-TREHALOSE	−
CYCLODEXTRIN	−	INULIN	−
D-GALACTOSE	−	D-GLUCOSE	+
GLYCOGEN	−	D-RIBOSE	−
myo-INOSITOL	−	PUTRESCINE assimilation	−
METHYL-A-D-GLUCOPYRANOSIDE acidification	−	GROWTH IN 6.5% NaCl	−
ELLMAN	−	KANAMYCIN RESISTANCE	−
METHYL-D-XYLOSIDE	−	OLEANDOMYCIN RESISTANCE	−
ALPHA-MANNOSIDASE	−	ESCULIN hydrolysis	+
MALTOTRIOSE	−	TETRAZOLIUM RED	−
Glycine ARYLAMIDASE	−	POLYMIXIN-B RESISTANCE	−

Key: + = positive, − = negative.

**Table 4:** Determination of MIC of pyraclostrobin against different tested pathogens.

Pathogens	MIC (µg/mL)	Standards (µg/mL)	DMSO (Control)
*B. cereus*	3.12	0.8	−
*S. aureus*	1.6	0.9	−
*E. coli*	12.5	1.6	−
*P. aeruginosa*	25	3.12	−
*S. flexneri*	6.25	1.6	−
*C. glabrata*	3.12	0.8	−

Key: − = No activity.

## Data Availability

Data are available upon request.
